# Comparison of qPCR and culture methods for group B Streptococcus colonization detection in pregnant women: evaluation of a new qPCR assay

**DOI:** 10.1186/s12879-018-3208-4

**Published:** 2018-07-05

**Authors:** J. A. Carrillo-Ávila, J. Gutiérrez-Fernández, A. I. González-Espín, E. García-Triviño, L. G. Giménez-Lirola

**Affiliations:** 1 Laboratorio de Microbiología, Universidad de Granada-Instituto de Investigación Biosanitaria de Granada, Av. de la Investigación n°11, 18071 Granada, Spain; 2 Laboratorio de Microbiología, Universidad de Granada, Hospital General Virgen de las Nieves- Instituto de Investigación Biosanitaria de Granada, Granada, Spain; 3 Hospital Materno Infantil de Jaén, Avenida de Ejército Español s/n, 23007 Jaén, Spain; 4 College of Veterinary Medicine, Iowa State University, Ames, Iowa, USA

**Keywords:** Molecular diagnostic, PCR, Group B *Streptococcus*, GBS colonization, Neonatal infection

## Abstract

**Background:**

*Streptococcus* Group B (GBS) colonization in pregnant women is the most important risk factor for newborn disease due to vertical transmission during delivery. GBS colonization during pregnancy has been implicated as a leading cause of perinatal infections. Traditionally, pregnant women are screened for GBS between 35 and 37 weeks of gestation. However, antenatal culture-based screening yields no information on GBS colonization status and offers low predictive value for GBS colonization at delivery. Numerous assays have been evaluated for GBS screening in an attempt to validate a fast and efficient method. The aim of this study was to compare bacteria isolation by culture and two qPCR techniques, targeting *sip* and *cfb* genes, respectively, for detecting colonizing GBS.

**Methods:**

Cultures – the gold-standard technique, a previous qPCR technique targeting the *sip* gene, and a new proposed qPCR assay targeting the *cfb* gene were evaluated as diagnostic tools on 320 samples.

**Results:**

Considering cultures as the gold standard, the evaluated qPCR method detected 75 out of 78 samples, representing a sensitivity of 93.58% (95% confidence interval (CI), 90.89–96.27) and specificity of 94.62% (95% CI, 91.78–97.46). However, an additional analysis was performed for true positives that included not only samples showing positives by culture but samples showing positive for both qPCR assays. The sensitivity and specificity were recalculated including these discrepant samples and a total of 89 samples were considered as positive, giving a prevalence of 27.81%. With this new analysis, the qPCR targeting the *cfb* gene showed a sensitivity of 95.5% (95% CI, 88.65–98.59) and specificity of 99.13% (95% CI, 96.69–99.97).

**Conclusions:**

The new qPCR method is a sensitive and specific assay for detecting GBS colonization and represents a valuable tool for identifying candidates for intrapartum antibiotic prophylaxis. Cultures should be retained as the reference and the routine technique because of its specificity and cost analysis ratio, but it would be convenient to introduce PCR techniques to check negative culture samples or when an urgent detection is required to reduce risk of infection among infants.

## Background

Group B *Streptococcus* (GBS) is a common commensal bacteria of gastrointestinal and vaginal flora with reported carriage rates ranging from 4 to 40% [1]. Colonization during pregnancy has been implicated as a leading cause of severe neonatal infections, including sepsis, pneumonia, and meningitis [2, 3]. Vertical transmission to the newborn occurs during labor via fetal aspiration of infected amniotic fluid or during passage through the birth canal [4]. Due to this vertical transmission, GBS infection is the a important cause of neonatal morbidity and mortality in the United States [5, 6].

Determination of infection at the time of delivery is essential for neonatal vertical transmission prevention [7], because some women are intermittent carriers of GBS and the rate of GBS colonization may vary during pregnancy [8]. The predictive value of antenatal screening decreases if it is performed more than a few weeks before delivery [9]. Neonatal infections can be prevented in most cases by providing intrapartum antibiotic prophylaxis to the colonized mother [10]. However the use of antibiotic prophylaxis on the basis of risk assessment leads to unnecessary treatment in many women [11].

Different methods are used to detect GBS, mainly culture and nucleic acids amplification methods [12]. Since 2002, the Center for Disease Control and Prevention (CDC) published guidelines for the prevention of perinatal GBS disease, recommending routine culture for all pregnant women between 35 and 37 weeks of gestation [3, 10]. The current CDC gold-standard method for GBS detection is rectovaginal sample incubation in a selective broth medium followed by subculture on a blood agar plate [3]. However, negative culture results have been observed in some women whose infants subsequently develop GBS infection [10]. The culture method for GBS detection has many disadvantages: the sensitivity is limited, a large turnaround time is needed, requiring up to 36 to 72 h before results can be reported [3], the lack of information on the GBS colonization status of some women at delivery, and the low predictive value of antenatal culture findings for GBS colonization at delivery [13, 14].

More sensitive and faster methods for detecting GBS colonization would help to obviate the need for prenatal screening to identify GBS, as well as reducing postpartum complications and severe infections in infants and avoiding the unnecessary use of antibiotic prophylaxis in women who are not colonized [15]. Nucleic acids amplification assays for detection of GBS colonization in pregnant women at delivery have shown good sensitivity and specificity [14, 15]. In the present study, a new nucleic acids amplification method over a *cfb* gene fragment has been developed and evaluated. The results are compared with a previously described method that uses the *sip* gene as a target [16] and with culturing as the current gold-standard method [17].

## Methods

### The study

A prospective study (November 2013–July 2015) was carried out on 320 clinical samples collected from pregnant women at the Andalucía public health system hospital network. The women included in the sample had not received antibiotic treatment the week prior to sample acquisition, and they did not show contraindication to vaginal examination. Paired vaginal/rectal swabs were used simultaneously to obtain rectovaginal GBS samples from consenting women between 35 and 37 weeks of gestation at a routine antenatal screening. Samples were transported in Stuart’s medium and were stored at 4 °C for a maximum of 48 h until examination. One swab was used for the GBS microbiological culture and the other was used for GBS qPCR testing. Collected swabs for culturing and qPCR testing, respectively, were stored at 2–8 °C and − 80 °C until the evaluation. The specific objective of this study was to compare culturing and the two qPCR techniques, targeting *sip* and *cfb* genes respectively, for detecting GBS colonization.

### Data analysis

In a primary analysis, culturing was considered the gold-standard technique for detecting GBS. Sensitivity and specificity data for cultures and both qPCR techniques are expressed in reference to the culture results. An additional analysis was performed for true positives that included not only samples showing positives by culture but samples showing positive for both qPCR assays. The cut-off for both qPCR techniques was established in 35 cycles.

### Specimen collection

The recommended specimen for either of the techniques considered here is a dual swab collected from the vaginal area followed by the rectal area, as previously described [3]. The swabs were then placed into the transport container (see above).

### Culture and identification of GBS

GBS cultures were performed according to the guidelines of the Spanish Society for Clinical Microbiology and Infectious Diseases [17]. For specimen enrichment, samples were inoculated in Todd Hewitt broth containing colistin and nalidixic acid (BD, Cat 296,266) to inhibit growth of enterobacteria and other microbiota present in the genital and gastrointestinal tract. After 18–24 h of enrichment at 37 °C in aerobiosis, the broth-enriched cultures were subcultured in Granada medium (BD, Cat 257,079) in anaerobiosis for up to 48 h. Cultures were examined for the presence of orange to red colonies as a presumptive identification of GBS.

### DNA extraction

The swabs that were previously collected and frozen at − 80 °C for PCR testing were thawed to carry out DNA extraction. The Amies transport medium swab (CM0425, Oxoid, Basingstoke, UK) was resuspended in 500 μl of 10 mM Tris-EDTA, pH 7.4. The swabs were manually rotated for 20 s inside the 1.5 ml tube and DNA was extracted using a QIAamp DNA mini kit (Qiagen, Valencia, CA) following the manufacturer’s instructions. The DNA was eluted in 200 μl of Qiagen kit AE buffer and stored at − 20 °C until use. An aliquot of 10 μl of the supernatant was used for PCR testing.

### Quantitative PCR methods

#### Quantitative PCR targeting the *sip* gene (PCR-A)

A fragment (78 bp) of the *sip* gene was amplified following the method described by Bergh et al. [16] with minor modifications [7]. Real time PCR was performed in a 7500 Applied Biosystems thermocycler (Applied Biosystems, MA, USA) following recommended protocol [7, 16]. Positive and negative controls were included in each run; a dilution corresponding to 10^3^ bacterial-DNA genome copies of *Streptococcus agalactiae* strain 2603 V/R was used as positive control and sterile water was added instead of DNA template as a negative control.

#### Quantitative PCR targeting the *cfb* gene (PCR-B)

A fragment (99 bp) of the *cfb* gene that codified for a diffusible extracellular protein called cAMP factor was amplified. Different *cfb* gene sequences from different GBS strains obtained from GenBank (X72754, HF952105.1, CP013202.1, HG939456.1, X72754.1, CP011326.1, CP007570.1, CP012419.2, CP012419.2, CP012419.2, CP010319.1, CP006910.1, CP007570.1, CP007631.2, HF952104.1, CP010867.1) were aligned and analyzed using Unipro UGENE 1.24 software [18] to identify highly conserved regions with the oligonucleotide design. We also used this program to identify homologous *cfb* gene sequences available in databases from *S. pyogenes* (AF079502), *S. uberis* (U34322), *S. canis* (AF488802), and *S. faecalis* (29374661) to rule out non-specific amplification. No homology was found with non-GBS species. The oligos selected were: 5’-GAAACATTGATTGCCCAGC-3′ and 5′.-AGGAAGATTTATCGCACCTG-3′. The Taqman probe was FAM 5’-CCATTTGATAGACGTTCGTGAAGAG-3’ BHQ-1. Real-time PCR was performed in a 7500 Applied Biosystem thermocycler using the following final concentrations: 2.5 mM C_2_Mg, 0.5 μM of each primer, and 0.25 μM Taqman probe. Positive and negative controls were included in each run; a dilution corresponding to 10^3^ bacterial genomes of *S. agalactiae* strain 2603 V/R was used as positive control. Sterile water was added instead of DNA template as negative control. The following PCR program was used: 94  °C (5 min), 40 cycles of 92  °C (10 s), 58 °C (10 s), and 72 °C (10 s).

#### Sequencing of discrepant samples

A discrepant result was defined as a result obtained with both PCR methods that did not correlate with the culture results. The discrepant results were resolved using bidirectional sequence analysis of the GBS *sip* and *cfb* genes [19]. The same oligonucleotides used for amplification that were described previously were used for fragment sequencing (GENYO, Granada, Spain). Sequences were analyzed using Unipro UGENE 1.24 software and Online Standard Nucleotide Blast.

### Sample size

A total of 320 samples collected from individual patients were included in this study. The optimal sample size was determined using the program “Power and size calculation” V3.1.2. [20] setting α error probability at 0.05 and power (1 − β error probability) at 0.95%, and based on an estimated SGB prevalence of 12–20% previously reported in Spain [17, 21]. Taking into account the expected prevalence aforementioned under the established conditions, the estimated minimum sample size was established between 163 (for a 12% prevalence) and 246 (for a 20% prevalence).

### Statistical analysis

The results from the qPCRs targeting *sip* or *cfb* were compared to the culture results. The performance characteristics, including sensitivity and specificity, were calculated using standard methods. The 95% confidence intervals (CI) were calculated using XLSTAT software (Addinsoft).

## Results

A total of 320 dual rectovaginal swabs were evaluated. Bacterial isolation by culture was considered the gold standard for GBS diagnosis (100% specificity is assumed) [17, 20]. Among the 78 samples indicated positive by culture, 75 and 73 samples were positive by PCR-A and PCR-B, respectively. A detailed diagram of the results is show in Table 1. The detection rate among PCR methods was 96.15% (95% CI, 94.04–98.26) for qPCR-A and 93.58% (95%, CI 90.89–96.27) for qPCR-B. However, no significant differences (*p* > 0.05) in the proportion of qPCR-positive samples detected by both qPCR methods were observed. Data for sensitivity and specificity are described in Table 2. The overall proportion of positive specimens [26.88% (95% CI, 22.31–31.99)] detected by both qPCRs was greater than that of the culture method [24.38% (95% CI, 19.98–29.37)].

**Table 1:** Comparative results between PCR-A (*sip* gene), PCR-B (*cfb* gene) considering culture as gold standard

		qPCR - A *(sip* gene)			qPCR - B *(cfb* gene)		
		Positive	Negative	Total	Positive	Negative	Total
Culture	Positive	75	3	78	73	5	78
	Negative	11	231	242	13	229	242
	Total	86	234	320	86	234	320

**Table 2:** Sensitivity and specificity for both qPCR techniques considering culture as gold standard

	Sensitivity	Specificity
qPCR-A	75/78 (96,15%)	231/242 (95,45%)
	(95% CI 94,04-98,26)	(95% CI 92,82-98,08)
qPCR-B	73/78 (93,58%)	229/242 (94,62%)
	(95% CI 90,89-96,27)	(95% CI 91,78-97,46)

In the present study, 11 samples tested positive by both PCR tests but negative by culture. Although culture is the reference technique for GBS detection, we considered samples in this group to be true positives, so positive results by culture (regardless of whether they are positive or not by any of the PCRs) and/or both PCR techniques (although negative by culture) were considered to be true positives (89 samples). Results with respect to this standard are given in Table 3. A GBS prevalence of 27.81% (95% CI 22.14–31.86) was obtained, and considering power samples sizes parameters defined previously (α = 0.05 and 1 − β = 0.95%) a minimum size of 309 samples was considered necessary to obtain statistically significant results. The sensitivity and specificity values obtained with culture and both qPCR methods are summarized in Table 4. Although five samples that tested positive by culture were negative by at least one of the PCR tests, 100% specificity was assumed for GBS culture. Some discrepancies were found between the three evaluated techniques. Samples found positive by either of the evaluated PCR techniques were sequenced to discard non-specific amplification. Discrepancies and the possible causes are presented in Table 5. Two samples were positive only by culture, and 11 samples were positive by both PCR tests and negative by culture. What’s more, two samples were positive by culture and PCR-A but negative by PCR-B, while one sample was positive by culture and PCR-B but negative by PCR-A. Finally, two samples were only positive by PCR-B.

**Table 3:** Comparative results between PCR-A (*sip* gene), PCR-B (*cfb* gene) and culture considering as real positives, samples positive by culture and/or both qPCR methods

		Culture + qPCR consensus (*n* = 89)		
		Positive	Negative	Total
Culture	Positive	78	6	84
	Negative	11	225	236
qPCR-A	Positive	86	0	86
	Negative	3	231	234
qPCR-B	Positive	85	2	87
	Negative	4	229	233
Total				320

**Table 4:** Sensitivity and specificity for culture and both qPCRs taking into account the new standard defined

	Sensitivity	Specificity
Culture	78/89 (87,64%)	100%
	(95% CI 80,8-94,48)	
qPCR-A	86/89 (96,62%)	231/231 (100%)
	(95% CI 90,14-99,26)	
qPCR-B	85/89 (95,5%)	230/231 (99,13%)
	(95% CI 88,65-98,59)	(95% CI 96,69-99,97)

**Table 5:** Resume of discrepancies between the three evaluated techniques

Number of discordant samples (N)	Culture	qPCR-A	qPCR-B
2	P	N	N
2	P	P	N
1	P	N	P
11	N	P	P
2	N	N	P

Positive results and the negative ones are indicated with P and N respectively

## Discussion

Since the publication of the CDC guidelines for the prevention of perinatal GBS disease in 2002, the incidence of neonatal infections has decreased more than 60% [22]. Several published studies have demonstrated the usefulness of culture-based [23, 24] and PCR-based methods [4, 25, 26] for detecting GBS. Traditionally, the culture method has been used as the common reference method, including in the Andalusian Sanitary System. However, the culture method may not be absolutely effective for GBS identification [27]. The use of a sensitive and accurate PCR method would provide the best means of GBS detection [28, 29]. Numerous qPCR procedures have been developed in recent years, and different genes have been selected as targets for the specific amplification of GBS, including the *cfb* gene, which encodes for cAMP factor and is the most frequently used [15, 30]; *cyl*E gene [7]; *dltR* gene [1]; *sip* gene [16]; *scpB* gene [31]; and C-protein gene [32].. Even commercial methods based on amplification techniques such as Xpert GBS (Cepheid, Sunnyvale, CA, USA) have appeared, helping to establish molecular techniques for GBS detection [33].

In the present study, a total of 320 vaginal/rectal swabs were tested by two qPCR methods. A new oligo selection, not previously used by other authors, was performed for *cfb* gene amplification. All samples were also tested by selective culture methods [17]. A specificity of 100% was assumed for bacterial cultures as the gold-standard method; the specificity of PCR-A and PCR-B was calculated to be 95.45 and 94.62%, respectively. Sensitivity was 96.15 and 93.58% for PCR-A and PCR-B, respectively.

Among samples that were negative by culture methods, 3.44% (11/320) were indicated to be positive by both qPCR methods (targeting *sip* and *cfb*). Therefore, non-specific amplification or contamination were unlikely to be the cause of this discrepancy. If we only consider the samples that were indicated positive by culture, we lose positives detected by PCR but not by culture. Traditionally, these results have been considered false PCR positives, but really, they are false culture negatives and must be considered for greater strength [7]. These results may indicate that culture may not be absolutely effective in the detection of GBS [22, 33]. The sensitivity and specificity were recalculated including these discrepant samples. A total of 89 samples that tested positive by culture (regardless of whether they are positive or not by any of the PCRs) and/or positive by both PCR techniques (although negative by culture) were considered true positives, giving an overall detection rate of 27.81%. The GBS detection rated reported in this study was slightly higher compared to previous studies in Spain [17, 21], but in agreement with the SGB prevalence reported in different studies performed in Europe [7, 34, 35]. According to previous studies, the detection rate for GBS ranged between 10 and 35% [4, 7, 22, 36]. The high variation in detection rates may be explained by differences in prevalence among different populations, differences in culture techniques and protocols, or differences in prevalence over time [37]. A diagnostic sensitivity of 96.62 and 95.50% was obtained for PCR-A and PCR-B, respectively, while culture sensitivity was reduced to 87.64%. The diagnostic specificity was estimated as 100% for both culture and PCR-A and as 99.13% for PCR-B. Based on these results, and in accordance with other studies, it can be concluded that qPCR techniques are more sensitive than culture methods [16, 22, 27, 33, 38, 39]. Different studies have compared PCR detection of the GBS *cfb* gene to broth culture and have reported sensitivities between 86.7 and 100% and specificities between 95.9 and 100% [13, 33]. Some authors have reported sensitivity values between 94 to 97% for an *sip* gene end-point PCR [15, 40]. Real-time PCR assays in pregnant women that targeted the *cfb* gene or *ptsI* gene in GBS have shown sensitivities of 45 to 100% compared to culture methods [13, 15, 41–44].

We observed discrepant results in 18 samples among the three evaluated tests. Specifically, two samples tested positive only by culture. As it is assumed that culture has a specificity of 100%, those samples were considered as false negatives by qPCR, although the samples were not checked by another technique to resolve discrepancies. These false negatives obtained by PCR were reported in samples with low colony growth (one colony observed in culture), which is probably below the detection limit of PCR techniques [33, 45]. Moreover, the use of a double swab collection (one processed for culture and the other for PCR) may cause variability in sensitivity between paired samples near the detection limit. Finally, another possible reason for these false negative PCR results may be the fact that in the present study the recommendations of the CDC for the optimization of DNA extraction have not been followed, which constitutes a study limitation [46]. A second group of discrepant samples, considered true positives, included three samples that were weak positives by culture (one or two colonies per plate) and weak positives by only one qPCR. A third group of discrepant samples included 11 samples testing positive via both qPCRs methods but negative by culture. As per previous discussion, the two qPCR methods used in this study were based on different target genes, so non-specific amplification was unlikely to occur and the specificity was confirmed by sequencing. Therefore, those 11 samples were considered as false negatives by culture. A possible explanation for those negative culture samples is loss of bacterial viability during specimen collection and/or transport, where the nonviable bacterial DNA remained available for amplification [47]. Also, it must be considered that the selective culture medium used in this study cannot detect non-hemolytic GBS colonies, which are estimated to constitute up to 4% of GBS infections [17]. A third explanation for the false culture negatives might be high density of recto-vaginal flora present in the culture that might cover up or inhibit GBS growth even when using selective broth medium [22, 33, 38, 48]. Finally two samples were only positive for PCR-B, which was confirmed by sequencing. Both weak positive samples appeared in cycles > 32 and could be due to a contamination event or to a higher sensitivity of PCR-B, although these samples were considered as negative in the statistical analyses performed, according to the standards established in the study.

In conclusion, different methods can be used for GBS detection in rectovaginal swabs from pregnant women. The new qPCR method evaluated in the present study may be a rapid diagnostic alternative that is easy to use as routinely diagnostic method. The method choice depends on technical aspects (lab expertise and lab equipment) or clinical aspects (preterm delivery, maternal fever during labor, or history of GBS disease in previous infants). Although qPCR techniques are more expensive than culture, they have been shown to have higher sensitivity and are faster than culture. Different authors have studied the cost of GBS culture screening versus PCR testing. Taking into account that the lower sensitivity of GBS culture methods results in unnecessary intrapartum antibiotic prophylaxis, an increase in hospital stay expenses, and early-onset GBS disease not detected by culture, they concluded that the final cost for both techniques was similar [49]. Considering the results of the present study, in which 11 samples (3.44% of total) that were positive for GBS were not detected by the culture method, introduction of amplification techniques in the diagnosis routine appears to be supported. Culture methods must continue to be the routine reference technique, but it would be convenient to introduce PCR techniques to at least verify culture negative samples. Commercial Xpert GBS proposes a fast and completely automated but expensive technique (1 to 2 h) with reduced manipulation that may be especially useful in pregnant women with ruptured membranes [33]. However, a considerable proportion of invalid or erroneous results (10.8 to 19%) have been described due to the presence of mucus or feces that inhibits the PCR and blocks the microfluidic channel in the cartridge [14, 50]. The test described in this paper, which has a turn-around time of 2 h, is mainly presented as a routine test for pregnant woman in the diagnosis routine, although it could also be used with patients in a relatively critical state, such as ruptured membranes at delivery, as long as there is availability of qPCR thermocycler in the hospital. PCR is easy to perform and easy to automate; however, the future improvement and automatization of DNA extraction procedures, would facilitate the implementation of amplification techniques for diagnosis.

## Conclusions

The determination of GBS infection at the time of delivery is essential for prevention of neonatal vertical transmission. A combined strategy based on bacteria culture technique and nucleic acid amplification techniques would be more effective than a single diagnostic method to avoid the appearance of false negatives, and the consequent increase in GBS infections in neonates. The new PCR technique described in this manuscript allow for a rapid and effective detection of GBS in carriers pregnant women. The introduction of molecular tools does not imply an increase in diagnostic costs, since their greater sensitivity reduces unnecessary intrapartum antibiotic prophylaxis, and reduces both hospital stays and costs.

## Abbreviations

CDC: Center for Disease Control and Prevention
cfb gene: complement factor B gene
GBS: Group B *Streptococcus*
sip gene: surface immunogenic protein
